# Exploration of various roles of hypoxia genes in osteosarcoma

**DOI:** 10.1038/s41598-022-17622-0

**Published:** 2022-10-31

**Authors:** Jimin Ma, Ziming Guo, Xuefei Yang, Yakun Zhu

**Affiliations:** Department of Orthopedics, Fuyang Hospital of Anhui Medical University, 99 Huangshan Road, Fuhe Modern Industrial Park, Yingzhou District, Fuyang, 236000 Anhui Province China

**Keywords:** Cancer, Immunology

## Abstract

Osteosarcoma is a primary malignant tumor that often metastasizes in orthopedic diseases. Although multi-drug chemotherapy and surgical treatment have significantly improved the survival and prognosis of patients with osteosarcoma, the survival rate is still very low due to frequent metastases in patients with osteosarcoma. In-depth exploration of the relationship between various influencing factors of osteosarcoma is very important for screening promising therapeutic targets. This study used multivariate COX regression analysis to select the hypoxia genes SLC2A1 and FBP1 in patients with osteosarcoma, and used the expression of these two genes to divide the patients with osteosarcoma into high-risk and low-risk groups. Then, we first constructed a prognostic model based on the patient's risk value and compared the survival difference between the high expression group and the low expression group. Second, in the high expression group and the low expression group, compare the differences in tumor invasion and inflammatory gene expression between the two groups of immune cells. Finally, the ferroptosis-related genes with differences between the high expression group and the low expression group were screened, and the correlation between these genes was analyzed. In the high-risk group, immune cells with higher tumor invasiveness, macrophages M0 and immune cells with lower invasiveness included: mast cell resting, regulatory T cells (Tregs) and monocytes. Finally, among genes related to ferroptosis, we found AKR1C2, AKR1C1 and ALOX15 that may be related to hypoxia. These ferroptosis-related genes were discovered for the first time in osteosarcoma. Among them, the hypoxia gene FBP1 is positively correlated with the ferroptosis genes AKR1C1 and ALOX15, and the hypoxia gene SLC2A1 is negatively correlated with the ferroptosis genes AKR1C2, AKR1C1 and ALOX15. This study constructed a prognostic model based on hypoxia-related genes SLC2A1 and FBP1 in patients with osteosarcoma, and explored their correlation with immune cells, inflammatory markers and ferroptosis-related genes. This indicates that SLC2A1 and FBP1 are promising targets for osteosarcoma research.

## Introduction

Osteosarcoma is a primary malignant tumor that often metastasizes in orthopedic diseases^1^. It often occurs in adolescents and is characterized by the malignant proliferation of primary mesenchymal stem cells leading to the malignant deposition of osteoid^2^. Although multi-drug chemotherapy and surgical treatment have significantly improved the survival and prognosis of patients with osteosarcoma, because patients with osteosarcoma often metastasize, their survival rate is still very low^3^. In-depth exploration of the relationship between multiple influencing factors in osteosarcoma is very important for screening promising therapeutic targets.

Hypoxia plays an important role in many cancers. Hypoxia is an important reason for radiotherapy tolerance, metastasis and poor prognosis of many malignant tumors^4^. At present, researchers have linked hypoxia and immune-related genes to verify the prognostic characteristics related to hepatocellular carcinoma. This prognostic feature is helpful for the personalized treatment of patients^5^. In addition, CD8 T cells activated by cancer immunotherapy promote specific lipid peroxidation in the process of tumor cell ferroptosis, and the increase of ferroptosis in tumor cells will contribute to the anti-tumor effect of immunotherapy^6^. Scientists have concluded that ferroptosis is related to carcinogenesis based on general human epidemiological data, specific disease data and animal research data^7^.

Therefore, in this study, we screened out the hypoxia genes SLC2A1 and FBP1 in patients with osteosarcoma through multivariate COX regression analysis, and used the expression of these two genes to divide patients with osteosarcoma into high-risk and low-risk groups. Then, we first constructed a prognostic model based on the patient's risk value to compare the survival difference between the high expression group and the low expression group. Secondly, in the high expression group and the low expression group, the differences in tumor infiltration of immune cells and the expression differences of inflammatory genes between the two groups were compared. Finally, screen out the ferroptosis-related genes that are different in the high-expression group and the low-expression group, and analyze the correlation among these genes. Therefore, in this study, we constructed a prognostic model based on hypoxia-related genes SLC2A1 and FBP1 in patients with osteosarcoma, and explored its correlation with immune cells, inflammatory markers and ferroptosis-related genes. This suggests that SLC2A1 and FBP1 are promising research targets in osteosarcoma.

## Results

### Hypoxia gene screening

**Figure 1:**
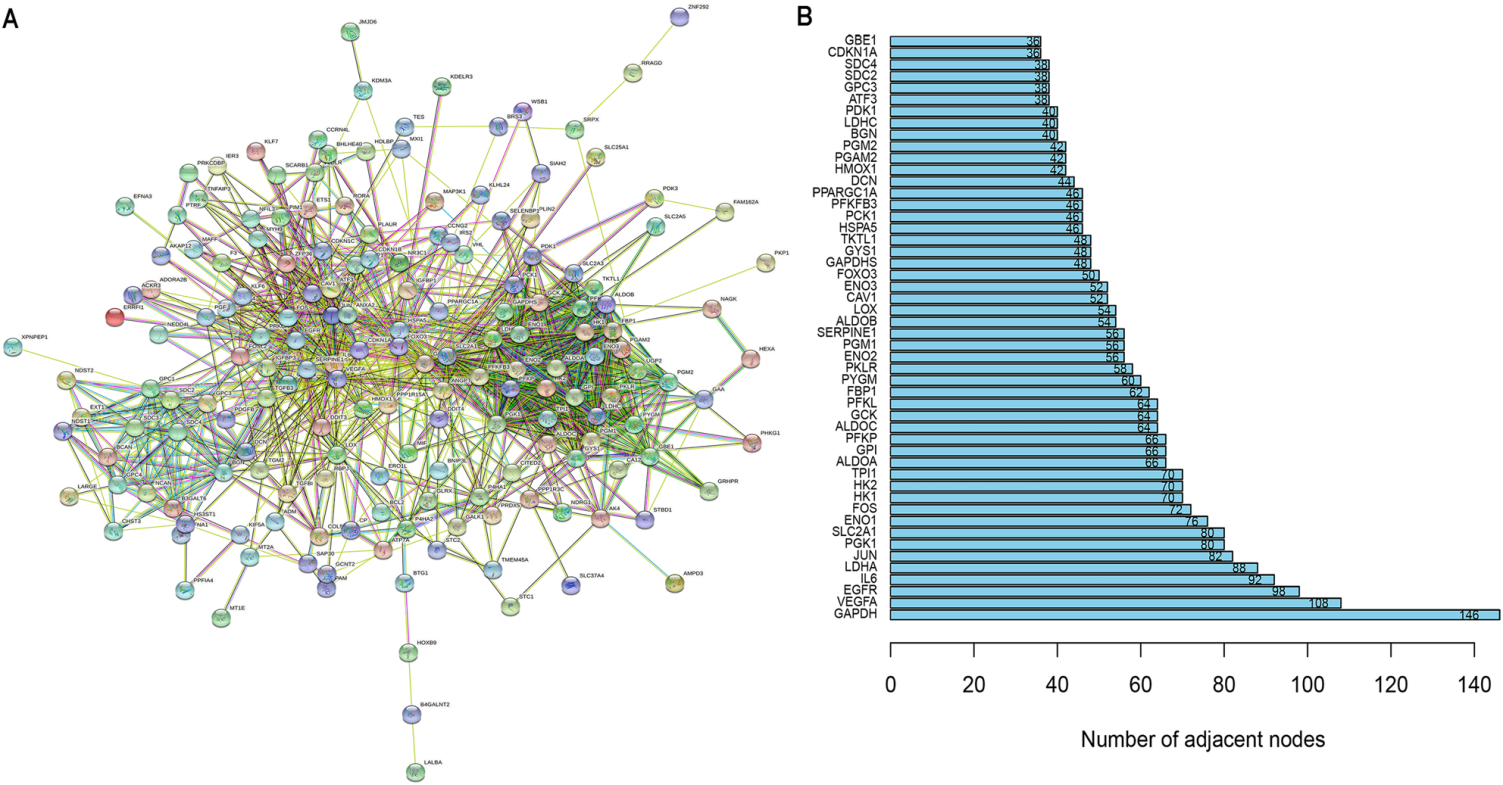
Screening hypoxia genes. (**A**) The protein interaction network diagram of hypoxia gene. (**B**) Histogram of the top fifty hypoxia genes.

On the STRING online website, we import the hypoxia gene set into the STRING online website to draw a protein interaction network (Fig. 1A), and draw a histogram to show the top 50 hypoxia genes (Fig. 1B).

### COX regression analysis

**Figure 2:**
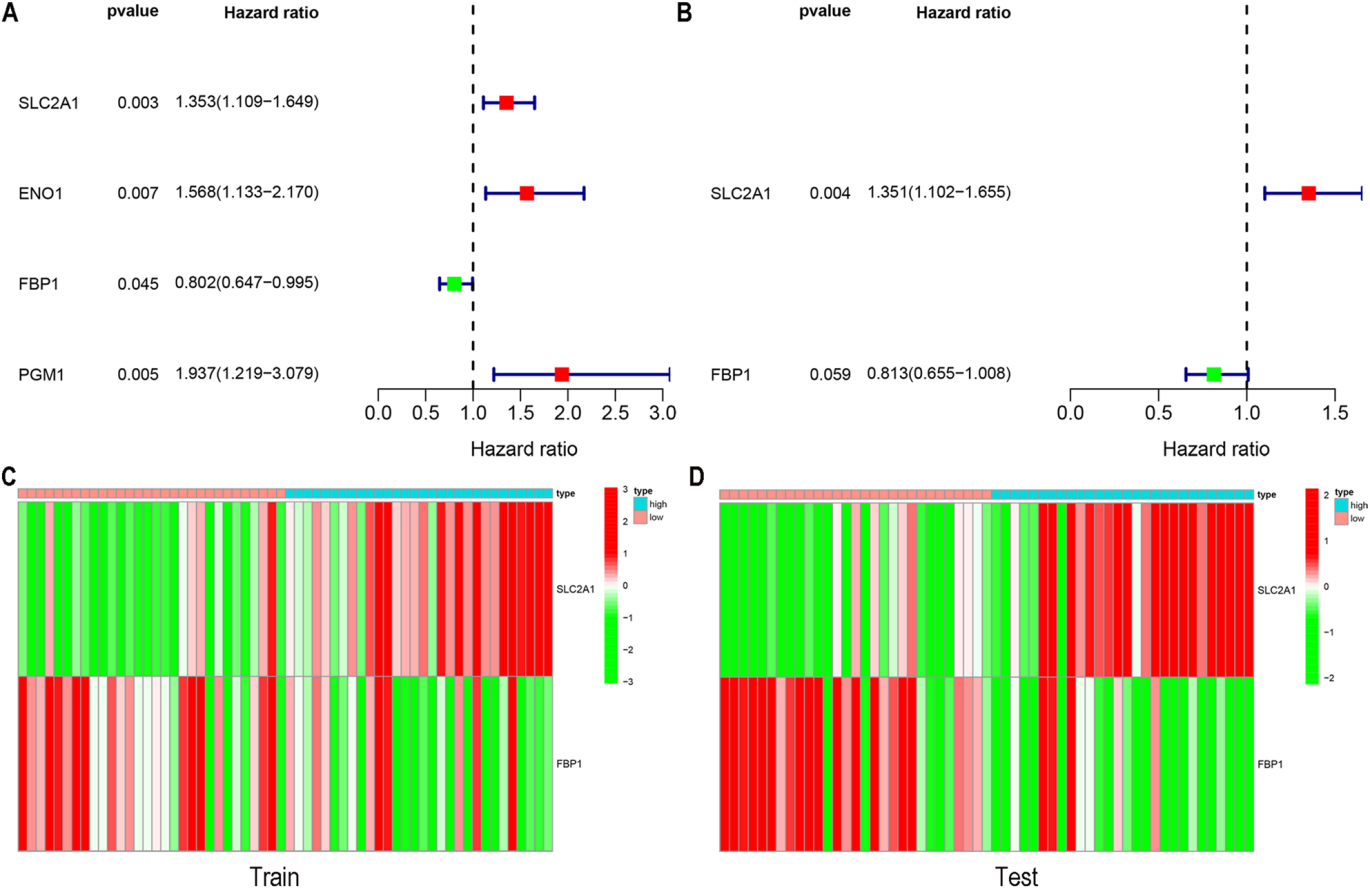
COX regression analysis (**A**&**B**) Single-factor COX regression analysis and multi-factor COX regression analysis select high-risk genes. (**C-D**) Draw two heat maps of the genes selected by the multivariate COX regression analysis to show the expression level of the genes in the train set and test set.

It has been found that hypoxia-related genes play an important role in osteosarcoma^8,9^. Therefore, we analyzed the relationship between the first 50 hypoxia genes and survival in patients with osteosarcoma. The results of univariate Cox regression analysis showed SLC2A1, ENO1, FBP1 and PGM1 (Fig. 2A), while multivariate COX regression analysis showed that SLC2A1 and FBP1 could indicate the prognostic risk of patients in osteosarcoma (Fig. 2B). We randomly divided the osteosarcoma samples into two groups, the training and the testing sets. Finally, in both sets, we created heatmaps to show the expression levels of SLC2A1 and FBP1(Fig. 2C,D).

### Build a prognostic model

**Figure 3:**
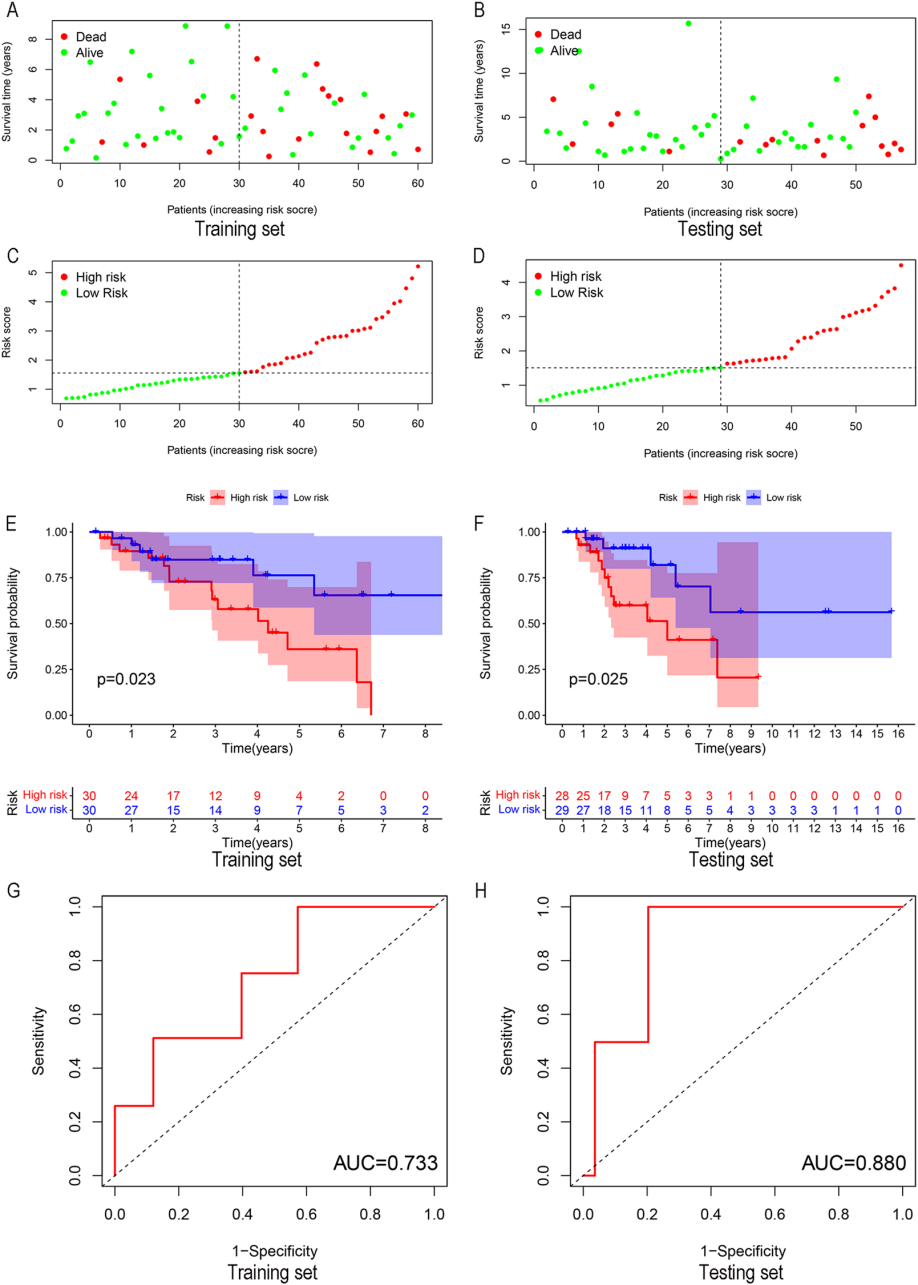
Constructing a prognostic model. (**A-D**) According to different risk values, patients with osteosarcoma are divided into high-risk groups and low-risk groups. (**E-F**) Construct a prognostic model between patients in the high-risk group and the low-risk group. The results show that patients in the low-risk group have a better prognosis. (**G-H**) Draw ROC curve to evaluate the accuracy of the prognostic model.

The expression levels of SLC2A1 and FBP1 in different samples were calculated in the training and the testing sets, the samples were quantified into different risk values, and the samples were divided into high-risk groups and low-risk groups according to the risk values (Fig. 3A–D). We then compared the survival analysis of patients in the high- and low-risk groups (Fig. 3E,F), and the results showed that the survival time was better in the low- and medium-risk groups in both groups (Training set: *p* = 0.023, Testing set: *p* = 0.025). The ROC curve indicated that the prognostic model had good accuracy (Training set: AUC = 0.733, Testing set: AUC = 0.880) (Fig. 3G,H).

### GSEA of hypoxia gene in osteosarcoma samples

**Figure 4:**
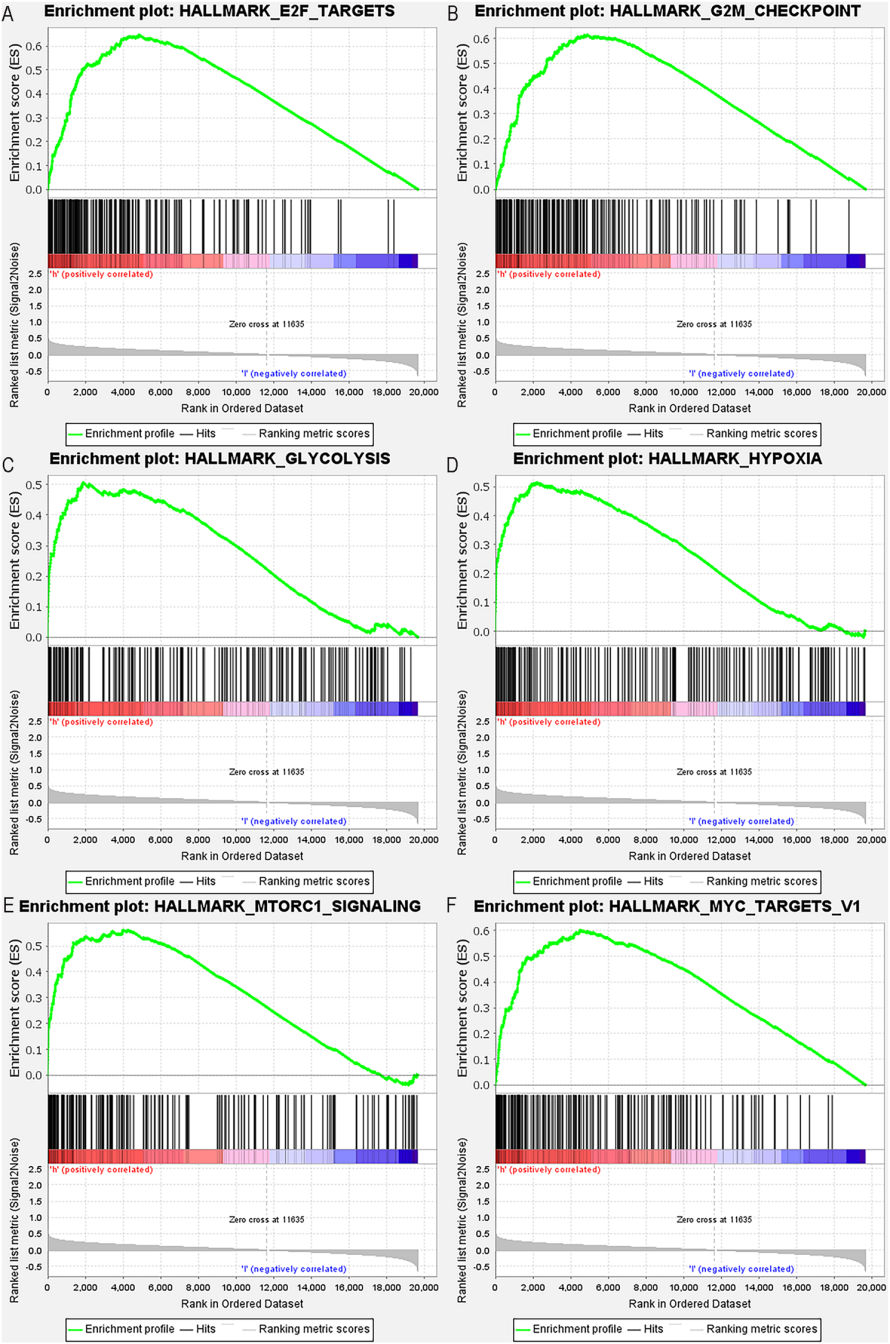
Enrichment plots of hypoxia-related gene sets from gene set enrichment analysis (GSEA). GSEA results show that the gene sets are in (**A**) E2F_TARGETS (**B**) G2M_CHECKPOINT, (**C**) GLYCOLYSIS (**D**) HYPOXIA (**E**) MTORC1_SIGNALING and (**F**) MYC_TARGETS_V1 is rich in differences in high-risk phenotypes.

To detect the pathway enrichment of the two genes SLC2A1 and FBP1 in the sample, we implemented GSEA. In the high expression group, we showed the first six enriched pathway terms (Fig. 4A–F): E2F_TARGETS, G2M_CHECKPOINT, GLYCOLYSIS, HYPOXIA, MTORC1_SIGNALING and MYC_TARGETS_V1.

### Differences in immune cell infiltration between the high-risk group and low-risk group

**Figure 5:**
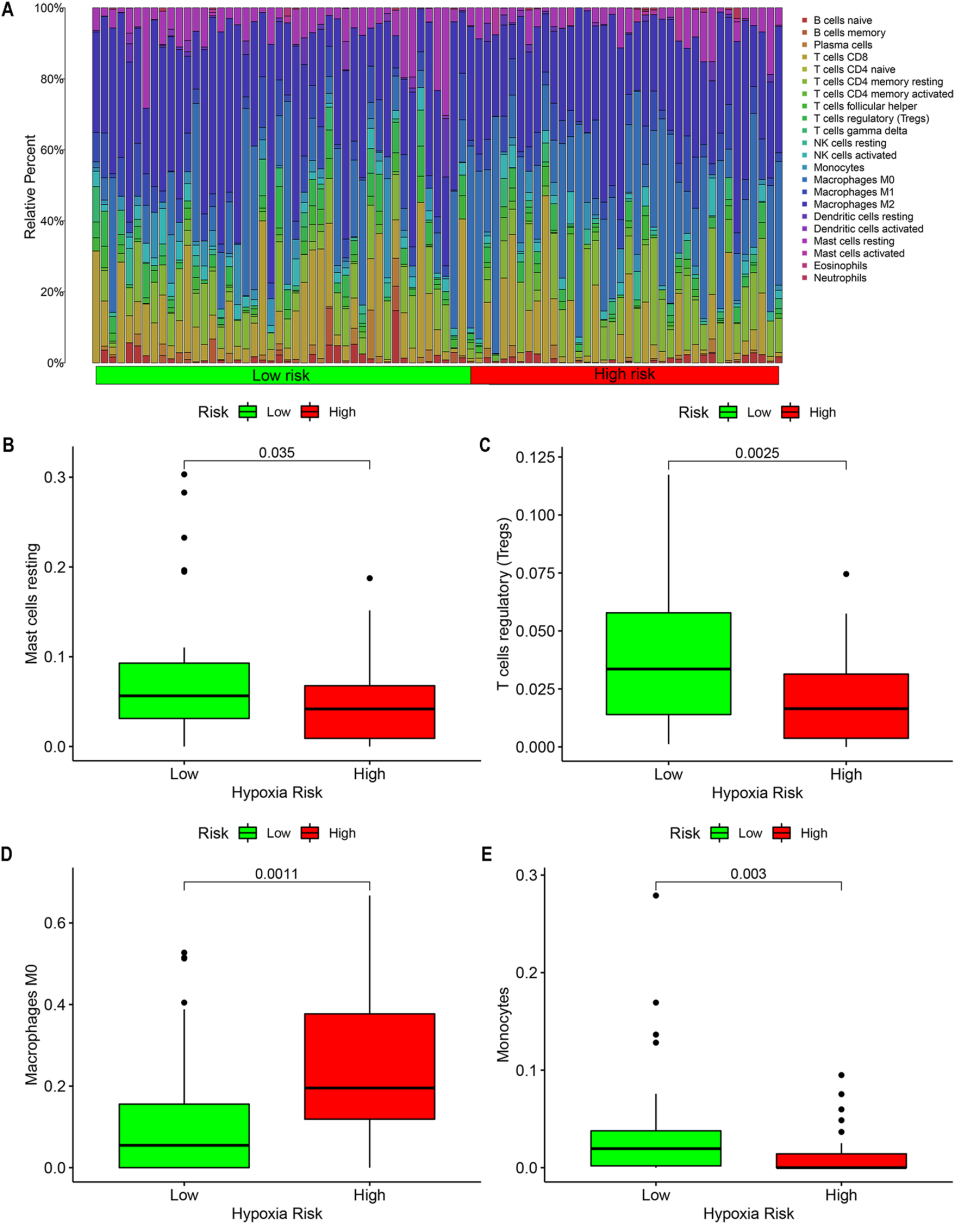
The difference in immune cell infiltration between the high-risk group and the low-risk group. (**A**) Heat map quantifying the degree of immune cell infiltration in the sample. (**B**-**E**) There are immune cells with different infiltration in the high-risk group and the low-risk group.

The immune cell infiltration in the two samples was quantified, and a heat map was drawn to show the difference in the infiltration of 22 immune cells in the two samples (Fig. 5A). Then, draw a box plot of the immune cells with differences in the two groups (Fig. 5B–E). The results showed that in the high-risk group, immune cells with higher tumor infiltration Macrophages M0 (Fig. 5D), and immune cells with lower infiltration include: Mast cells resting, T cells regulatory (Tregs) and Monocytes (Fig. 5B,C,E).

### Differences in immune gene expression between the high-risk group and low-risk group

**Figure 6:**
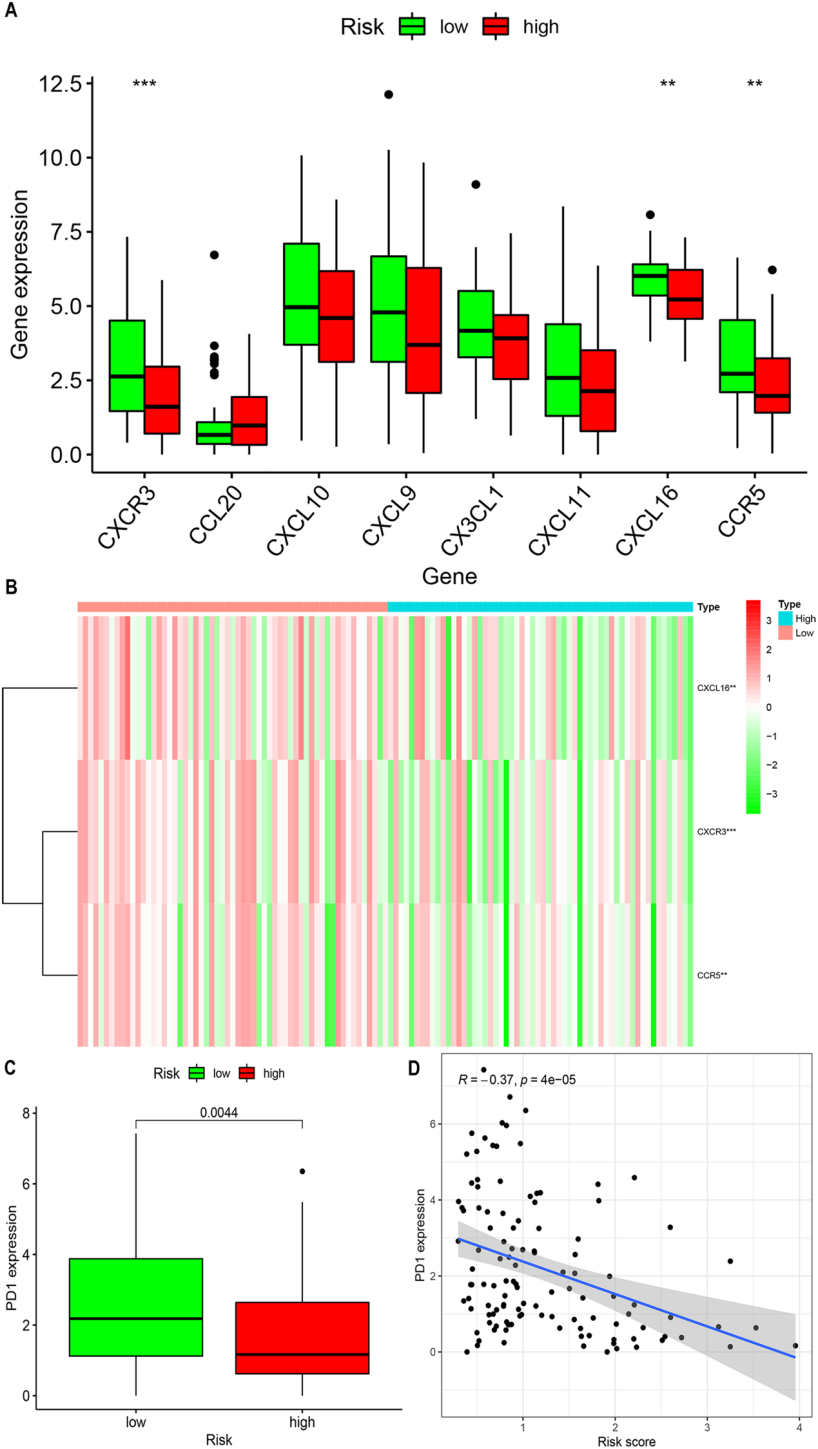
Differences in the expression of immune genes and PD1 between the high-risk group and the low-risk group. (**A**,**B**) The expression levels of CXCL16, CXCR3 and CCR5 are significantly different in the two groups of samples, and the expression levels are higher in the low expression group. (**C**) Box plot of PD1 expression levels in the high expression group and the low expression group. (**D**) Scatter plot of the correlation between PD1 and patient risk score.

After screening the tumor-infiltrating differential immune cells in the high expression group and the low expression group, further search for differentially expressed immune genes. To compare CXCR3, CCL20, CXCL10, CXCL9, CX3CL1, CXCL11, CXCL16 and CCR5 in the high expression group and low expression group, draw a box plot to compare (Fig. 6A). The results showed that the expression levels of CXCL16, CXCR3 and CCR5 were significantly different in the two groups of samples, and the expression levels were higher in the low expression group (Fig. 6B). Finally, we compared the difference in PD1 expression between the two groups, as well as the difference in PD1 expression and patient risk scores (Fig. 6C,D). The box plot shows that in the low-risk group, the expression of PD1 is higher than that of the high-expression group (*p* = 0. 0044). The correlation analysis results show that PD1 expression is negatively correlated with the patient's risk score (R =  − 0. 37, *p* = 0. 00,005).

### The relationship between hypoxia gene and ferroptosis gene in osteosarcoma

**Figure 7:**
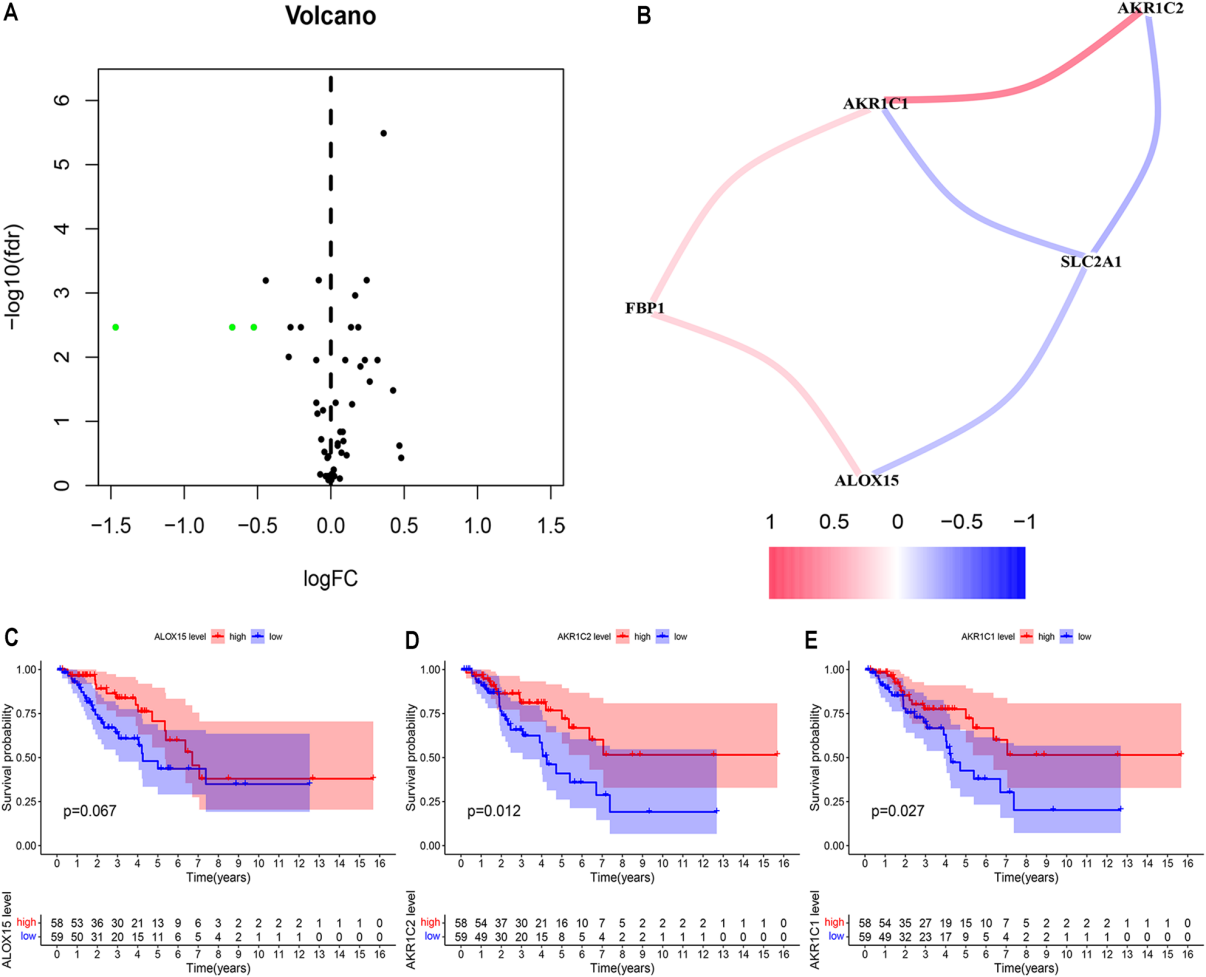
The relationship between hypoxia genes and ferroptosis genes. (**A**) The volcano map of the ferroptosis gene differentially expressed in the high-risk group and the low-risk group. (**B**) The correlation between two hypoxia genes and three ferroptosis-related genes in osteosarcoma is analyzed. The hypoxia gene FBP1 is positively correlated with the ferroptosis genes AKR1C1 and ALOX15, and the hypoxia gene SLC2A1 is related to the ferroptosis-related genes AKR1C2, AKR1C1 and ALOX15. There is a negative correlation. (**C-E**) Survival curves of AKRIC2, AKRIC1 and ALOX15 in patients with osteosarcoma.

Because in many studies, hypoxia genes and ferroptosis genes are closely related^5,10^. In the end, we will explore the differences in ferroptosis-related genes between the high-risk group and the low-risk group. First, through differential analysis, we screened out three differentially expressed ferroptosis genes: AKRIC2, AKRIC1 and ALOX15 (Fig. 7A). Subsequently, the correlation between two hypoxia genes and three ferroptosis-related genes in osteosarcoma was analyzed (Fig. 7B). The hypoxia gene FBP1 was positively correlated with the ferroptosis genes AKR1C1 and ALOX15, and the hypoxia gene SLC2A1 and the ferroptosis gene AKR1C2 were positively correlated. AKR1C1 and ALOX15 are negatively correlated. Finally, the survival curves of the three ferroptosis-related genes AKRIC2, AKRIC1 and ALOX15 in osteosarcoma patients were drawn (Fig. 7C,D,E).

## Discussion

The bone microenvironment is composed of bone marrow and mineralized extracellular matrix^11^. The bone marrow microenvironment is fertile soil so that osteosarcoma often metastasizes^11,12^. The hypoxic tumor microenvironment functions as the dominant driving force for cancer progression, drug resistance, development of metastatic potential, and overall poor clinical outcomes^13,14^. The relationship between hypoxia-related genes and the occurrence and development of osteosarcoma has received more and more attention. It is particularly important to screen out promising hypoxia genes in osteosarcoma.

The purpose of this study is to screen out meaningful hypoxia genes in osteosarcoma, and to explore the correlation of these genes with tumor immune microenvironment and ferroptosis-related genes. Hypoxia is not only an important tumor microenvironmental marker in the pathogenesis of breast cancer patients, but also is related to the increased risk of death in breast cancer patients^15^. Hypoxia is an unfavorable factor in the occurrence of cervical cancer, and the expression of hypoxia-related genes can be used to identify biomarkers of aggressive hypoxic tumors^16^. Hypoxia microenvironment is also a feature of patients with pancreatic cancer. Hypoxia can induce the expression of tumor-related malignant behavior genes^17^. In osteosarcoma, hypoxia is associated with chemotherapy resistance and reduced survival in patients with osteosarcoma^18^. Specifically, hypoxia can regulate the immune microenvironment of tumors. For example, hypoxia can promote the recruitment of innate immune cells, inhibit the differentiation of adaptive immune cells and reduce the function of adaptive immune cells^19^.

When a hypoxic area appears in the tumor, the tumor can also restore part of the blood and nutrient supply^20^, but hypoxia can trigger the expression of hypoxia inducible factor (HIF), which in turn promotes tumor metastasis^21^. In the immune microenvironment of osteosarcoma, tumor-infiltrating immune cells (TIICs) and inflammatory mediators can help the proliferation and spread of osteosarcoma cells^22^. Ferroptosis provides a treatment strategy to overcome the apoptosis resistance and multidrug resistance of solid tumors, and synergistically strengthen the chemotherapy of hypoxic tumors^23^.

At present, there are few reports on the relationship between hypoxia-related genes in osteosarcoma in terms of immunity and ferroptosis. Therefore, in this study, we first screened two prognostic-related hypoxia genes, Solute Carrier Family 2 Member 1 (SLC2A1) and Fructose-Bisphosphatase 1 (FBP1), in osteosarcoma. SLC2A1 and FBP1 have been reported to be closely related to cancer in bladder cancer^24^, lung cancer^25^, thyroid cancer^26^ and breast cancer^27^. In osteosarcoma, we constructed a prognostic model for SLC2A1 and FBP1, and the prognosis of the patient can be predicted based on the risk score of expression. There are also significant differences in tumor immune cell infiltration between the high-risk group and the low-risk group. In the high-risk group, immune cells with higher tumor invasion, Macrophages M0, and immune cells with lower invasion include: Mast cells resting, T cells regulatory (Tregs) and Monocytes. In fact, hypoxia can shape and induce specific macrophage phenotypes, thereby promoting the malignant development of tumors, because hypoxia can promote immune evasion, angiogenesis, tumor cell survival and metastatic spread^28^. Hypoxia can also affect the integrin-mediated adhesion of mast cells to fibronectin, and the location of mast cells in tumors is related to their functions^29^. Finally, among ferroptosis-related genes, we found Aldo–Keto Reductase Family 1 Member C2 (AKR1C2), Aldo–Keto Reductase Family 1 Member C1 (AKR1C1) and Arachidonate 15-Lipoxygenase (ALOX15) that may be related to hypoxia. These ferroptosis-related genes have been discovered for the first time in osteosarcoma. Among them, the hypoxia gene FBP1 is positively correlated with the ferroptosis genes AKR1C1 and ALOX15, and the hypoxia gene SLC2A1 is negatively correlated with the ferroptosis genes AKR1C2, AKR1C1 and ALOX15.

## Conclusion

We screened out the promising hypoxia genes SLC2A1 and FBP1 in osteosarcoma, and the patient risk score based on gene expression is related to the degree of tumor infiltration of multiple immune cells. Finally, we discovered for the first time the possible relationship between hypoxia genes and ferroptosis-related genes, laying a foundation for an in-depth study of the internal mechanism.

## Methods

### Data sources

The mRNA sequencing and clinical data of 119 osteosarcoma patients were downloaded from the TCGA portal (http://tcga.cancer.gov/dataportal)^30^. The list of hypoxia-associated genes was obtained from the hallmark-genes set of the Molecular Signatures Database^31^.

### Hypoxia gene screening

Import the hypoxia-genes set into STRING online website^32^, to construct protein interaction network. The top 50 genes with the largest number of connection points between genes were screened out, and the histogram was drawn using the “ggplot2” package in the R software.

### Hypoxia genes associated with osteosarcoma

Single-factor Cox analysis and multi-factor Cox analysis were used to screen hypoxia genes related to the survival of patients with osteosarcoma, and were displayed in forest diagrams and heatmap. R packages “survival” and the “pheatmap” packages were used for forest map and heatmap.

### Construction of the prognostic model

We randomly divided the osteosarcoma samples into two groups, the training and the testing sets. The selected hub genes are calculated based on the expression level in each osteosarcoma sample to calculate the risk scores and divided into high-risk groups and low-risk groups. The “Survival” and “survminer” package is used to calculate the relationship between the sample risk score and survival time, and to draw the survival curve. Use the “survival ROC” package to draw the receiver operating characteristic curve (ROC), and Area Under Curve (AUC) was defined as the area under the ROC curve to show the accuracy of the prognostic model.

### Gene set enrichment analysis

The Gene Set Enrichment Analysis (GSEA) can conduct an in-depth analysis by focusing on the relationship between gene sets in biological functions and regulatory genes^33^. Kyoto encyclopedia of genes and genomes (KEGG) pathway analyses were performed on the hub gene set using GSEA (version: 4. 1. 0), and the first six most relevant enrichment pathways were screened out. c2. cp. kegg. v6. 2. symbols. Gmt was set to the reference gene set and permutations = 1000 for each analysis. |normalized enrichment score (NES) |≥ 1. 0, *p*-value ≤ 0. 05, and False Discovery Rate (FDR) *q*-value ≤ 0. 25 were considered statistically significant.

### The relationship between hypoxia genes and immune cells

To further explore other possible roles of selected hypoxia genes in patients with osteosarcoma, we pooled all samples and created a histogram to compare immune cell infiltration between high- risk and low-risk groups according to patients' risk scores difference. Use the “CIBERSORT” package^34^ to evaluate the degree of immune cell infiltration in each patient with osteosarcoma, and repeat 100 times to obtain reliable results. Finally, we use the “ggplot2” package to draw box plots of the screened out differential immune cells to show the differences.

### The relationship between risk score and immune marker

The Wilcoxon rank sum test was used to compare the expression differences of different immune genes in the high-risk group and the low-risk group. Use the “ggplot2” package and the “pheatmap” package to draw heat maps and box plots to show the different immune markers. To evaluate the relationship between the risk score and immunosuppressive agents, we drawn a boxplot to compare the differential expression of PD1 in the high-risk and the low-risk groups, and a scatter plot was drawn to show the correlation between PD1 expression and the risk score.

### The relationship between hypoxia gene and ferroptosis gene

In the samples of the high-risk group and the low-risk group, we used the “limma” package to analyze the differential expression of ferroptosis-related genes and draw a volcano map (|log FC|> 0. 5, *p* < 0. 05). Then, we use the “igraph” package and “reshape2” package to analyze the correlation between ferroptosis-related differential genes and hypoxia hub genes. “Survival” and “survminer” packages were used to draw survival curves of ferroptosis differential genes.

### Consent to participate

Informed consent was obtained from all individual participants included in the study.

## Data Availability

The data of this study are from the TCGA database.

## Abbreviations

TCGA: The Cancer Genome Atlas
KEGG: Kyoto encyclopedia of genes and genomes
GO: Gene Ontology
DEGs: Differentially expressed genes
ssGSEA: Single sample gene set enrichment analysis
TME: Tumor microenvironment

## Code availability

All analyses were performed using R version 3. 6. 3.
